# Reactogenicity of Simultaneous COVID-19 mRNA Booster and Influenza Vaccination in the US

**DOI:** 10.1001/jamanetworkopen.2022.22241

**Published:** 2022-07-15

**Authors:** Anne M. Hause, Bicheng Zhang, Xin Yue, Paige Marquez, Tanya R. Myers, Casey Parker, Julianne Gee, John Su, Tom T. Shimabukuro, David K. Shay

**Affiliations:** 1 CDC COVID-19 Response Team, Centers for Disease Control and Prevention, Atlanta, Georgia; 2 Rollins School of Public Health, Emory University, Atlanta, Georgia

## Abstract

**Importance:**

COVID-19 and seasonal influenza vaccines are essential in preventing respiratory infections and their potentially severe complications. Simultaneous administration of vaccines is efficient and may improve coverage with each vaccine. However, the safety of simultaneous administration of COVID-19 and influenza vaccines has not been well described.

**Objective:**

To evaluate adverse events and health impacts associated with simultaneously administered COVID-19 mRNA booster and seasonal influenza vaccines in the US population.

**Design, Setting, and Participants:**

In this retrospective cohort study, self-reported vaccine data were collected on days 0 to 7 after vaccination from September 22, 2021, through May 1, 2022, through v-safe, a voluntary smartphone-based monitoring system established by the Centers for Disease Control and Prevention. Participants were persons who voluntarily registered in v-safe following COVID-19 vaccination.

**Exposure:**

Receipt of simultaneously administered COVID-19 mRNA booster and seasonal influenza vaccines or COVID-19 mRNA booster alone.

**Main Outcomes and Measures:**

Local injection site and systemic reactions (eg, fatigue, headache, and myalgia) and health impacts reported by v-safe respondents in the week following COVID-19 mRNA booster vaccination. Adjusted odds ratios (aORs) were estimated for simultaneous administration compared with booster dose alone, controlling for sex, age, and week of vaccination.

**Results:**

Of a total of 981 099 persons aged 12 years or older registered with v-safe, simultaneous administration of COVID-19 mRNA booster and seasonal influenza vaccines was reported by 92 023 (9.4%) v-safe respondents; of these respondents, 54 926 (59.7%) were female, 36 234 (39.4%) were male, and sex was unknown for 863 (0.9%). In the week following vaccination, any systemic reactions were reported by 36 144 (58.9%) of 61 390 respondents who simultaneously received Pfizer-BioNTech booster and influenza vaccines and 21 027 (68.6%) of 30633 respondents who simultaneously received Moderna booster and influenza vaccines. Respondents who simultaneously received influenza and Pfizer-BioNTech booster vaccines (aOR, 1.08; 95% CI, 1.06-1.10) or influenza and Moderna booster vaccines (aOR, 1.11; 95% CI, 1.08-1.14) were slightly more likely to report any systemic reaction in the week following simultaneous vaccination than respondents who received only a COVID-19 mRNA vaccine booster.

**Conclusions and Relevance:**

In this study, compared with administration of COVID-19 mRNA booster vaccines alone, simultaneous administration of COVID-19 mRNA booster and seasonal influenza vaccines was associated with significant increases in reports of systemic reactions during days 0 to 7 following vaccination. These results may help better characterize the outcomes associated with simultaneously administered COVID-19 booster and influenza vaccines in the US population.

## Key Points

**Question:**

Are systemic reactions more common after simultaneous administration of COVID-19 mRNA booster and seasonal influenza vaccines than after COVID-19 mRNA booster alone?

**Findings:**

In this cohort study of self-reported data from 981 099 persons aged 12 years or older, simultaneous administration of a COVID-19 mRNA booster dose and an influenza vaccine was associated with 8% to 11% increases, respectively, in systemic reaction compared with COVID-19 mRNA booster alone. These differences were statistically significant.

**Meaning:**

Findings from this study suggest that simultaneous administration of COVID-19 mRNA booster and influenza vaccines may be associated with increased likelihood of systemic reactions.

## Teaser

This cohort study evaluates adverse events and health impacts reported to a smartphone-based safety surveillance system during days 0 to 7 after simultaneous COVID-19 mRNA booster and seasonal influenza vaccination in US adults.

## Introduction

COVID-19 vaccination is recommended by the Centers for Disease Control and Prevention (CDC) for all persons aged 5 years or older in the US.^1^ Current clinical guidance indicates that COVID-19 vaccines may be administered without regard to timing of other vaccines, including simultaneous administration of COVID-19 vaccine and other vaccines.^2^

The US Food and Drug Administration first expanded COVID-19 vaccine emergency use authorizations to include booster doses for adults aged 65 years or older and other high-risk populations (September 22, 2021, for Pfizer-BioNTech and October 20, 2021, for Moderna and Janssen); on November 19, 2021, emergency use authorizations were expanded to include all adults aged 18 years or older at least 6 months after completing a Pfizer-BioNTech or Moderna 2-dose primary series and at least 2 months after a single dose of Janssen vaccine.^3,4,5^ On December 9, 2021, the Pfizer-BioNTech emergency use authorization was expanded to include homologous booster doses for persons aged 16 years or older; on January 3, 2022, it was expanded to include children aged 12 years or older, and shorten the time to booster vaccination to 5 months.^3,4,5^

Authorization of COVID-19 vaccine booster doses coincided with the recommended period for seasonal influenza vaccination, increasing the likelihood of simultaneous administration of COVID-19 and influenza vaccines.^6^ To better characterize the safety of simultaneously administered COVID-19 booster and influenza vaccines in the US population, we evaluated adverse events and health impacts reported to v-safe, a smartphone-based safety surveillance system, during days 0 to 7 following vaccination during September 22, 2021, to May 1, 2022.

## Methods

The v-safe platform is a smartphone-based active safety surveillance system^7^ established by the CDC to collect information on health status among US residents after COVID-19 vaccination. This cohort study was reviewed by the CDC consistent with applicable federal law and CDC policy and was deemed not to be human participant research as defined in 45 CFR 46.102(l)(2); institutional review board review was not required. This report follows the Strengthening the Reporting of Observational Studies in Epidemiology (STROBE) reporting guideline for observational studies.

The v-safe platform is a voluntary surveillance system, and registered participants opt in to receive health surveys; no further consent was required. Following COVID-19 vaccination, persons may register with v-safe, indicate COVID-19 vaccine dose and any simultaneously administered vaccines (received during the same visit), and receive regularly scheduled text message reminders about completing online health surveys. During registration, persons indicate their age, race, and ethnicity; demographic variables were collected to evaluate how well the surveillance population represents the general US population. Health surveys sent in the first week postvaccination include questions about local injection site (pain, redness, swelling, or itching) and systemic reactions (chills, headache, joint pain, myalgia, fatigue, nausea, vomiting, diarrhea, abdominal pain, or rash) self-identified as mild, moderate, or severe and health impacts (whether the vaccine recipient was unable to perform normal daily activities, missed work or school, or received care from a medical professional [telehealth, outpatient, emergency department, or hospitalization]) (eMethods in the Supplement). Registrants may provide more information via free text about reactions or health impacts. A report indicating a medically attended event prompts contact by the CDC v-safe call center; completion of a vaccine adverse event reporting system report, if indicated, is encouraged.

The v-safe data collected during September 22, 2021, to May 1, 2022, were analyzed and described for v-safe respondents who simultaneously received COVID-19 mRNA booster and seasonal influenza vaccines or a COVID-19 mRNA booster vaccine alone and who completed at least 1 health survey during days 0 to 7 following vaccination. A booster dose was defined as a third dose of COVID-19 mRNA vaccine administered at least 5 months after dose 2, on or after the day on which booster doses were authorized for each manufacturer and age group. Excluded from the analysis were persons who (1) reported simultaneous administration of COVID-19 mRNA booster dose, influenza vaccine, and additional vaccine(s) (n = 2647); (2) reported a booster dose prior to its authorization (n = 26 960); or (3) reported a Moderna COVID-19 vaccine and were aged less than 18 years or Pfizer-BioNTech COVID-19 vaccine and were aged less than 12 years (n = 18). The odds of reporting any injection site or systemic reactions or health impacts following simultaneous administration of COVID-19 mRNA booster dose and influenza vaccines were compared with those following administration of the COVID-19 mRNA booster alone using a logistic regression model, adjusting for sex, age at vaccination, and week of vaccination. Statistical significance was set at α = .05 and 95% CIs were estimated. SAS software, version 9.4 (SAS Institute Inc) was used to conduct all analyses.

## Results

From September 22, 2021, to May 1, 2022, a total of 92 023 v-safe respondents reported simultaneous receipt of COVID-19 mRNA booster and seasonal influenza vaccines; 889 076 v-safe respondents reported receipt of a COVID-19 mRNA booster alone. Of respondents who simultaneously received COVID-19 mRNA booster and seasonal influenza vaccines, 54 926 (59.7%) were female, 36 234 (39.4%) were male, and sex was unknown for 863 (0.9%); 37 359 (40.6%) were aged 12 to 49 years, 23 760 (25.8%) were aged 50 to 64 years, 24 855 (27.0%) were aged 65 to 74 years, and 6049 (6.6%) were aged 75 years or older (Table 1).

**Table 1.** Demographic Characteristics of v-safe Respondents^a^

Characteristic	No. (%)			
	Simultaneous influenza and COVID-19 mRNA booster vaccine received^b^ (N = 92 023)		Only COVID-19 mRNA booster vaccine received (N = 889 076)	
	Pfizer-BioNTech (n = 61390)	Moderna (n = 30 633)	Pfizer-BioNTech (n = 466 439)	Moderna (n = 422 637)
Sex				
Female	36 717 (59.8)	18 209 (59.4)	293 245 (62.9)	267 029 (63.2)
Male	24 115 (39.3)	12 119 (39.6)	169 680 (36.4)	152 261 (36.0)
Unknown	558 (0.9)	305 (1.0)	3514 (0.8)	3347 (0.8)
Age range, y				
12-49^c^	24 036 (39.2)	13 323 (43.5)	187 310 (40.2)	144 262 (34.1)
50-64	15 406 (25.1)	8354 (27.3)	121 488 (26.1)	108 497 (25.7)
65-74	17 512 (28.5)	7343 (24.0)	120 736 (25.9)	131 121 (31.0)
≥75	4436 (7.2)	1613 (5.3)	36 905 (7.9)	38 757 (9.2)
Ethnicity^d^				
Hispanic/Latino	3798 (6.2)	2145 (7.0)	42 706 (9.2)	31 320 (7.4)
Not Hispanic/Latino	55 954 (91.2)	27 651 (90.3)	406 651 (87.2)	376 502 (89.1)
Unknown	1638 (2.7)	837 (2.7)	17 082 (3.7)	14 815 (3.5)
Race^d^				
American Indian or Alaska Native	326 (0.5)	155 (0.5)	1961 (0.4)	1922 (0.5)
Asian	2824 (4.6)	1269 (4.1)	38 160 (8.2)	25 692 (6.1)
Black	3019 (4.9)	1551 (5.1)	29 234 (6.3)	24 072 (5.7)
Native Hawaiian or Other Pacific Islander	122 (0.2)	54 (0.2)	1496 (0.3)	1005 (0.2)
White	51 920 (84.6)	25 845 (84.4)	361 219 (77.4)	344 592 (81.5)
Multiracial	1184 (1.9)	648 (2.1)	9513 (2.0)	7285 (1.7)
Other	990 (1.6)	572 (1.9)	13 550 (2.9)	9337 (2.2)
Unknown	1005 (1.6)	539 (1.8)	11 306 (2.4)	8732 (2.1)

^a^ Includes persons who completed at least 1 v-safe health check-in survey on days 0 to 7 after vaccination.

^b^ Simultaneous vaccination was defined as persons who received a COVID-19 mRNA booster vaccine and seasonal influenza vaccine during the same visit. A booster dose was defined as dose 3 of a COVID-19 mRNA vaccine administered ≥5 months after dose 2, on or after booster doses were authorized for each manufacturer and age group.

^c^ At the time of this analysis, the Moderna COVID-19 vaccine was authorized for use among persons aged ≥18 years and Pfizer-BioNTech COVID-19 booster dose was authorized for use among persons aged ≥12 years. Respondents aged <18 years indicating that they received Moderna COVID-19 vaccine and <12 years indicating that they received Pfizer-BioNTech COVID-19 booster dose were excluded from this analysis.

^d^ Registrants self-identify ethnicity and race; registrants may indicate their race to be “other.”

In the week after vaccination, systemic reactions were reported by 36 144 respondents (58.9%) who simultaneously received Pfizer-BioNTech COVID-19 booster and influenza vaccines and 21 027 respondents (68.6%) who simultaneously received Moderna COVID-19 booster and influenza vaccines (Table 2). Most reactions were reported as mild or moderate (Table 3) and were reported most frequently on the day following vaccination. The most frequently reported systemic reactions following simultaneous administration were fatigue, headache, and myalgia. Adjusted odds ratios (aORs) were estimated for simultaneous administration compared with booster dose alone, controlling for sex, age, and week of vaccination. Respondents who simultaneously received an influenza vaccine and either the Pfizer-BioNTech COVID-19 booster (aOR, 1.08; 95% CI, 1.06-1.10) or the Moderna COVID-19 booster (aOR, 1.11; 95% CI, 1.08-1.14) were slightly more likely to report any systemic reaction in the week following vaccination than respondents who received a COVID-19 mRNA booster alone (Table 4).

**Table 2.** Reactions Reported by v-safe Respondents^a^

Event	No. (%)			
	Simultaneous influenza and COVID-19 mRNA booster vaccine received^b^ (N = 92 023)		Only COVID-19 mRNA booster vaccine received (N = 889 076)	
	Pfizer-BioNTech (n = 61 390)	Moderna (n = 30 633)	Pfizer-BioNTech (n = 466 439)	Moderna (n = 422 637)
Any injection site reaction	39 818 (64.9)	22 800 (74.4)	298 529 (64.0)	302 947 (71.7)
Itching	4462 (7.3)	4066 (13.3)	35 116 (7.5)	55 302 (13.1)
Pain	38 151 (62.2)	21 615 (70.6)	285 016 (61.1)	284 825 (67.4)
Redness	5024 (8.2)	4652 (15.2)	36 642 (7.9)	61 020 (14.4)
Swelling	8609 (14.0)	7128 (23.3)	66 923 (14.4)	90 356 (21.4)
Any systemic reaction	36 144 (58.9)	21 027 (68.6)	274 539 (58.9)	274 107 (64.9)
Abdominal pain	2448 (4.0)	1558 (5.1)	20 855 (4.5)	20 077 (4.8)
Myalgia	20 376 (33.2)	13 353 (43.6)	155 768 (33.4)	167 511 (39.6)
Chills	10 092 (16.4)	7885 (25.7)	83 657 (17.9)	102 833 (24.3)
Diarrhea	3697 (6.0)	2041 (6.7)	26 279 (5.6)	25 141 (6.0)
Fatigue	27 153 (44.2)	16 170 (52.8)	203 368 (43.6)	206 828 (48.9)
Fever	11 205 (18.3)	8413 (27.5)	90 370 (19.4)	104 549 (24.7)
Headache	20 686 (33.7)	13 206 (43.1)	162 513 (34.8)	170 138 (40.3)
Joint pain	10 624 (17.3)	7594 (24.8)	85 608 (18.4)	97 333 (23.0)
Nausea	5887 (9.6)	4069 (13.3)	48 203 (10.3)	51 208 (12.1)
Rash	580 (0.9)	393 (1.3)	5314 (1.1)	6169 (1.5)
Vomiting	546 (0.9)	370 (1.2)	4635 (1.0)	4553 (1.1)
Any health impact^c^	11 658 (19.0)	8210 (26.8)	97 730 (21.0)	106 707 (25.3)
Unable to perform normal daily activities	9519 (15.5)	6811 (22.2)	80 427 (17.2)	90 671 (21.5)
Unable to work or attend school	4971 (8.1)	3561 (11.6)	40 645 (8.7)	41 481 (9.8
Needed medical care	510 (0.8)	258 (0.8)	4737 (1.0)	3911 (0.9)
Telehealth	157 (0.3)	78 (0.3)	1601 (0.3)	1278 (0.3)
Clinic	212 (0.4)	110 (0.4)	1718 (0.4)	1440 (0.3)
Emergency visit	67 (0.1)	37 (0.1)	711 (0.2)	519 (0.1)
Hospitalization	17 (0.03)	5 (0.02)	150 (0.03)	115 (0.03)

^a^ Percentage of enrollees who reported a reaction or health impact at least once during days 0 to 7 post-vaccination.

^b^ Simultaneous vaccination was defined as persons who received a COVID-19 mRNA booster vaccine and seasonal influenza vaccine during the same visit. A booster dose was defined as dose 3 of a COVID-19 mRNA vaccine administered ≥5 months after dose 2, on or after booster doses were authorized for each manufacturer and age group.

^c^ Any health impact was defined as persons who reported they were unable to perform normal daily activities, missed work or school, or received care from a medical professional because of new symptoms or conditions.

**Table 3.** Most Common Reactions Reported by v-safe Respondents^a^

Event^c^	No. (%)			
	Simultaneous influenza and COVID-19 mRNA booster vaccine received^b^ (N = 92 023)		Only COVID-19 mRNA booster vaccine received (N = 889 076)	
	Pfizer-BioNTech (n = 61 390)	Moderna (n = 30 633)	Pfizer-BioNTech (n = 466 439)	Moderna (n = 422 637)
Injection site pain	38 151 (62.2)	21 615 (70.6)	285 016 (61.1)	284 825 (67.4
Mild	24 989 (40.7)	12 252 (40.0)	178 894 (38.4)	167 510 (39.6)
Moderate	12 060 (19.6)	8378 (27.4)	95 377 (20.5)	104 754 (24.8)
Severe	1102 (1.8)	985 (3.2)	10 745 (2.3)	12 561 (3.0)
Fatigue	27 153 (44.2)	16 170 (52.8)	203 368 (43.6)	206 828 (48.9)
Mild	11 209 (18.3)	5636 (18.4)	80 911 (17.4)	76 066 (18.0)
Moderate	13 179 (21.5)	8391 (27.4)	99 576 (21.4)	106 321 (25.2)
Severe	2765 (4.5)	2143 (7.0)	22 881 (4.9)	24 441 (5.8)
Myalgia	20 376 (33.2)	13 353 (43.6)	155 768 (33.4)	167 511 (39.6)
Mild	8702 (14.2)	4894 (16.0)	61 869 (13.3)	62 529 (14.8)
Moderate	9813 (16.0)	6924 (22.6)	77 097 (16.5)	86 266 (20.4)
Severe	1861 (3.0)	1535 (5.0)	16 802 (3.6)	18 716 (4.4)
Headache	20 686 (33.7)	13 206 (43.1)	162 513 (34.8)	170 138 (40.3)
Mild	10 661 (17.4)	6094 (19.9)	80 113 (17.2)	80 883 (19.1)
Moderate	8432 (13.7)	5784 (18.9)	67 698 (14.5)	73 048 (17.3)
Severe	1593 (2.6)	1328 (4.3)	14 702 (3.2)	16 207 (3.8)

^a^ Percentage of enrollees who reported a reaction or health impact at least once during days 0 to 7 after vaccination.

^b^ Simultaneous vaccination was defined as persons who received a COVID-19 mRNA booster vaccine and seasonal influenza vaccine during the same visit. A booster dose was defined as dose 3 of a COVID-19 mRNA vaccine administered ≥5 months after dose 2, on or after booster doses were authorized for each manufacturer and age group.

^c^ Severity was self-reported as mild (symptoms noticeable, but not a problem), moderate (symptoms limit normal daily activities), or severe (symptoms make daily activities difficult or impossible).

**Table 4.** Reactions and Health Impacts Reported in v-safe Respondents

Reaction	Simultaneous influenza and COVID-19 mRNA booster vaccine received, aOR (95% CI)^a^^,^^b^ (N = 92 023)	
	Pfizer-BioNTech (n = 61 390)	Moderna (n = 30 633)
Any injection site reaction	1.10 (1.08-1.12)	1.05 (1.02-1.08)
Any systemic reaction	1.08 (1.06-1.10)	1.11 (1.08-1.14)
Any health impact^c^	0.99 (0.97-1.02)	1.05 (1.02-1.08)
Unable to perform normal daily activities	0.99 (0.97-1.01)	1.04 (1.01-1.07)
Unable to work or attend school	1.04 (1.01– 1.07)	1.08 (1.04-1.12)
Needed medical care	0.92 (0.84-1.01)	0.94 (0.83-1.07)

^a^ Includes persons who completed at least one v-safe health check-in survey on days 0 to 7 after vaccination. Odds ratios were adjusted for age at vaccination, sex, and week of vaccination.

^b^ Simultaneous vaccination was defined as persons who received a COVID-19 mRNA booster vaccine and seasonal influenza vaccine during the same visit. A booster dose was defined as dose 3 of a COVID-19 mRNA vaccine administered ≥5 months after dose 2, on or after booster doses were authorized for each manufacturer and age group.

^c^ Any health impact was defined as persons who reported they were unable to perform normal daily activities, missed work or school, or received care from a medical professional because of new symptoms or conditions.

Health impacts in the week after vaccination were reported by 11 658 respondents (19.0%) who simultaneously received Pfizer-BioNTech COVID-19 booster and influenza vaccines and 8210 respondents (26.8%) who simultaneously received Moderna COVID-19 booster and influenza vaccines. Inability to perform normal daily activities in the week following vaccination was reported by 9519 respondents (15.5%) who simultaneously received Pfizer-BioNTech COVID-19 booster and influenza vaccines and 6811 respondents (22.2%) who simultaneously received Moderna COVID-19 booster and influenza vaccines; fewer than 1% of respondents simultaneously administered vaccines required medical care, including 22 respondents (0.02%) who reportedly received care at a hospital. Information regarding reported hospitalizations (through a linked vaccine adverse event reporting system report, v-safe follow-up call, or v-safe free-text data) was available for 19 (86.4%) of the 22 respondents, including 10 (45.5%) who indicated hospitalization was unrelated or reported in error. Among the remaining respondents, reasons for hospitalization included excessive vomiting, supraventricular tachycardia, cardiac arrhythmia, chest discomfort, autoinflammatory disease flare, kidney infection, hypoxia, and stroke. Respondents who simultaneously received influenza and Pfizer-BioNTech COVID-19 booster vaccines were not more likely to report a health impact in the week following vaccination (aOR, 0.99; 95% CI, 0.97-1.02) than respondents who received a COVID-19 Pfizer-BioNTech COVID-19 booster alone; respondents who simultaneously received influenza and Moderna COVID-19 booster vaccines were slightly more likely to report any health impact in the week following vaccination (aOR, 1.05; 95% CI, 1.02-1.08) than respondents who received a Moderna COVID-19 booster alone.

## Discussion

The v-safe respondents who simultaneously received Pfizer-BioNTech or Moderna COVID-19 booster and seasonal influenza vaccines reported systemic reactions in the week after vaccination 8% to 11%, respectively, more frequently than did respondents who received a COVID-19 mRNA booster vaccine alone. Reactions after simultaneous vaccination or COVID-19 mRNA booster vaccination alone were usually mild. The phase 4 trial^8^ that included persons who simultaneously received dose 2 Pfizer-BioNTech COVID-19 and influenza vaccines did not identify any serious safety concerns. Hospitalization following either of these vaccine combinations was uncommonly reported to v-safe. Given the limited data collected by v-safe, it is not possible to determine whether hospitalization was related to vaccination unless specifically noted by the respondent.

Our findings are generally consistent with results from the phase 3 trial^9^ of simultaneous administration of an adjuvanted recombinant subunit vaccine for prevention of herpes zoster with substantial reactogenicity and quadrivalent inactivated influenza vaccine. Participants aged 50 years or older who received simultaneous vaccination reported systemic reactions more frequently (60.9%) than participants who received adjuvanted recombinant subunit herpes zoster vaccine (52.1%) or influenza vaccine (33.6%) alone; however, this difference was not considered clinically meaningful by Schwarz et al.^9^ Although we did not collect information on reactions after influenza vaccine alone, influenza vaccines in general are not particularly reactogenic. However, as reported by Falsey et al,^10^ some influenza vaccines appear to be more reactogenic for others; for example, among adults aged 65 years or older, local reactions were more frequently reported following high-dose influenza vaccine than standard-dose vaccine.

### Limitations

This analysis has limitations. The v-safe platform is a voluntary program, and, as a result, data might not be generalizable to the entire population receiving COVID-19 vaccines. The v-safe program relies on self-report of COVID-19 vaccine received; therefore, some misclassification of exposure is possible. The v-safe app collects information of solicited adverse events following COVID-19 vaccination and is not designed to detect rare unsolicited adverse events. Given the short interval between vaccination and submission of v-safe responses, recall bias should be minimal. To minimize the complexity of data collection for respondents, v-safe does not include information regarding influenza vaccine type. Some influenza vaccines are more reactogenic than others, especially those approved for older adults; however, our model controlled for age, and this adjustment may address the differential reactogenicity of influenza vaccines administered to older adults. In addition, it is possible that the small increases in reactogenicity found with simultaneous administration were due to residual confounding between those who received only a COVID-19 booster vs both COVID-19 and influenza vaccines.

## Conclusions

In this cohort analysis, initial safety findings from v-safe for simultaneously administered COVID-19 mRNA booster dose and seasonal influenza vaccines indicate that respondents who received simultaneous administration were slightly more likely to report systemic reactions in the week following vaccination than respondents who received COVID-19 mRNA booster alone. In both groups, most reactions reported in the week following vaccination were generally mild. These results may help better characterize the outcomes associated with simultaneously administered COVID-19 mRNA booster and influenza vaccines in the US population.

## Notes

Supplement. **eMethods.** V-safe Health Survey Sent During Days 0-7 Following Vaccination
