# Supplementary material for: Reactogenicity of Simultaneous COVID-19 mRNA Booster and Influenza Vaccination in the US
Source: JAMA Netw Open. 2022 Jul 15;5(7):e2222241. doi: 10.1001/jamanetworkopen.2022.22241 (PMC9287747; doi:10.1001/jamanetworkopen.2022.22241)
Supplement: Supplement. — eMethods. V-safe Health Survey Sent During Days 0-7 Following Vaccination [file jamanetwopen-e2222241-s001.pdf]

# Supplemental Online Content

Hause AM, Zhang B, Yue X, et al. Reactogenicity of simultaneous COVID-19 mRNA booster and influenza vaccination in the United States. *JAMA Netw Open.* 2022;5(7):e2222241. doi:10.1001/jamanetworkopen.2022.22241

**eMethods.** v-safe Health Survey Sent During Days 0-7 Following Vaccination

This supplemental material has been provided by the authors to give readers additional information about their work.

© 2022 Hause AM et al. *JAMA Network Open*.

**eMethods**

**v-safe Health Survey sent during Days 0-7 following Vaccination**

1. How are you feeling today?
   - Good
   - Fair
   - Poor

2. Since your vaccination, have you had a fever or felt feverish?
   - Yes
   - No

   (If Yes) Do you know your highest temperature reading from today?
   - Yes- in degrees Fahrenheit
   - Yes- in degrees Celsius
   - No- I don't remember the reading
   - No- I didn't take my temperature

   Enter your highest temperature reading from today (degrees Fahrenheit): ____________

   Enter your highest temperature reading from today (degrees Celsius): _______________

3. Have you had any of these symptoms at or near the injection site? Select all that apply.
   - Pain
   - Redness
   - Swelling
   - Itching
   - None

   (For each checked symptom) How would you rate your symptom?
   - Mild = you notice symptoms, but they aren't a problem
   - Moderate = symptoms that limit of your normal daily activities
   Severe = symptoms make normal daily activities difficult or impossible

4. Have you experienced any of these symptoms today? Select all that apply.
   - Chills
   - Headache
   - Joint pain
   - Muscle or body aches
   - Fatigue or tiredness
   - Nausea
   - Vomiting
   - Diarrhea
   - Abdominal pain
   - Rash, not including the immediate area around the injection site

© 2022 Hause AM et al. *JAMA Network Open*.

- None

(For each checked symptom) How would you rate your symptom?

- Mild = you notice symptoms, but they aren't a problem
- Moderate = symptoms that limit of your normal daily activities
- Severe = symptoms make normal daily activities difficult or impossible

5. Any other symptoms or health conditions you want to report______________________

6. Did any of the symptoms or health conditions you reported TODAY cause you to (select all that apply):
   - Be unable to work or attend school?
   - Be unable to do your normal daily activities?
   - Get care from a doctor or other healthcare professional?
   - None of the above

(If "Get care…" checked) What type of healthcare visit did you have? (check all that apply)

- Telehealth, virtual health, or email health consultation
- Outpatient clinic or urgent care clinic visit
- Emergency room or emergency department visit
- Hospitalization
- Other, describe: ____________________________________________________

© 2022 Hause AM et al. *JAMA Network Open*.
